# Combination of blockade of endothelin signalling and compensation of IGF1 expression protects the retina from degeneration

**DOI:** 10.1007/s00018-023-05087-x

**Published:** 2024-01-22

**Authors:** Naoya Shigesada, Naoya Shikada, Manabu Shirai, Michinori Toriyama, Fumiaki Higashijima, Kazuhiro Kimura, Toru Kondo, Yasumasa Bessho, Takuma Shinozuka, Noriaki Sasai

**Affiliations:** 1 Division of Biological Science, Nara Institute of Science and Technology, Ikoma, 630-0192 Japan; 2 Omics Research Center (ORC), National Cerebral and Cardiovascular Center, Suita, Osaka 564-8565 Japan; 3 Department of Biomedical Chemistry, School of Science and Technology, Kwansei Gakuin University, Sanda, 669-1337 Japan; 4 Department of Ophthalmology, Graduate School of Medicine, Yamaguchi University, Ube, 755-0046 Japan; 5 Division of Stem Cell Biology, Institute for Genetic Medicine, Hokkaido University, Sapporo, 060-0815 Japan

**Keywords:** Retinitis pigmentosa, Prominin-1, Insulin-like growth factor (IGF), Gliosis, Mammalian/mechanical target of rapamycin (mTOR), Adeno-associated virus (AAV), Single-cell RNA sequencing (scRNA-seq)

## Abstract

Retinitis pigmentosa (RP) and macular dystrophy (MD) cause severe retinal dysfunction, affecting 1 in 4000 people worldwide. This disease is currently assumed to be intractable, because effective therapeutic methods have not been established, regardless of genetic or sporadic traits. Here, we examined a RP mouse model in which the *Prominin-1* (*Prom1*) gene was deficient and investigated the molecular events occurring at the outset of retinal dysfunction. We extracted the Prom1-deficient retina subjected to light exposure for a short time, conducted single-cell expression profiling, and compared the gene expression with and without stimuli. We identified the cells and genes whose expression levels change directly in response to light stimuli. Among the genes altered by light stimulation, *Igf1* was decreased in rod photoreceptor cells and astrocytes under the light-stimulated condition. Consistently, the insulin-like growth factor (IGF) signal was weakened in light-stimulated photoreceptor cells. The recovery of *Igf1* expression with the adeno-associated virus (AAV) prevented photoreceptor cell death, and its treatment in combination with the endothelin receptor antagonist led to the blockade of abnormal glial activation and the promotion of glycolysis, thereby resulting in the improvement of retinal functions, as assayed by electroretinography. We additionally demonstrated that the attenuation of mammalian/mechanistic target of rapamycin (mTOR), which mediates IGF signalling, leads to complications in maintaining retinal homeostasis. Together, we propose that combinatorial manipulation of distinct mechanisms is useful for the maintenance of the retinal condition.

**Supplementary Information:**

The online version contains supplementary material available at 10.1007/s00018-023-05087-x.

## Introduction

Daily information from the environment is acquired mainly through visual transduction, and the loss of visual function causes severe impacts on the quality of life of individuals. Understanding the initiation and progression of retinal degeneration and implementing therapeutic methods are, therefore, of great interest [1].

Among several eye diseases, RP and MD are major disorders occurring in the retina. In many instances, degeneration begins in photoreceptors and/or retinal pigment epithelium (RPE) cells [1, 2], and subsequently spreads throughout the entire retina. One in 4000 people suffer from RP and MD [3–5], half of whom have genetic traits, and disorders are passed from ancestors to descendants in specific pedigrees. More than 60 genes have been identified as causative for RP and MD, and Prominin-1 (Prom1/CD133), the focus in this study, is one of such genes [6–10].

The *Prom1* gene encodes a pentaspan membrane protein and is highly expressed in the retina [8], testis and kidney at the tissue level [11]. Regarding subcellular localisation, PROM1 is present in the disc region of photoreceptor cells [8] and in the microvilli of the neuroepithelial cell membrane [12, 13]. The physiological function of Prom1 is difficult to anticipate due to the low similarity between its polypeptide sequence and other proteins. Nevertheless, it is highly probable that Prom1 is involved in the morphogenesis of membranes and the secretion of vesicles, based on several observations on membrane processes, including the specific localisation of cholesterol [14], the development of extensions [15], and the formation of extracellular vesicles [13, 14, 16].

Mutations in the *Prom1* gene have been reported in some families where people suffer from RP and MD [8]. The mice attenuated with the *Prom1* gene (*Prom1−/−*; *Prom1*-KO) well recapitulate the symptoms found in human patients, making the *Prom1*-mutant mice a suitable animal model to investigate the progression of retinal degeneration [9, 10]. *Prom1*-mutant mice can be born normally; however, the retinas of the mutant mice start to degenerate immediately after the eyes open [9]. *Prom1*-mutant mice exhibit severe photoreceptor loss and failure of autophagy of RPE cells [17]; therefore, drusen, the accumulation of lipids and proteins that should have been removed, accumulates around the RPE cell layer. Light stimulation is a major trigger of the retinal phenotype [6]; *Prom1*-KO mice exhibit programmed cell death and progressive thinning of the outer nuclear layer (ONL) of the retina [6].

In our previous study, we conducted high-throughput expression profiling and identified genes whose expression levels are altered in the retina at the earliest step of retinal degeneration [6, 18]. Although no significant change was found in any genes, except for *Prom1* itself, in the 2-week-old *Prom1*-KO retinas compared to the wild-type ones, more than 1000 genes were altered in their expression compared to the wild-type retinas at 3 weeks after birth. This analysis showed that endothelin signalling was markedly activated in the *Prom1*-KO retina, which induced *glial fibrillary acidic protein* (*Gfap*) gene expression to activate glial cells. Although the activation of glial cells leads to the production of neurotrophic factors that may act to rescue photoreceptor cell survival and function [19–21], excess glial activation leads to gliosis, where glial cells clump together and become a spatial obstacle in the retina. In addition, the endothelin signal induces stenosis of the retinal vessels by vasoconstriction [22], and the delivery of substances from the blood to the surrounding cells, especially the photoreceptor cells, diminishes. Treatment with antagonists targeting endothelin receptors can alleviate the phenotype, suggesting that excess endothelin signalling negatively affects the retina, and blockade of the endothelin signal partially recovers the phenotypes observed during retinal degeneration. The activation of the endothelin signal and subsequent gliosis has also been shown in other mutant mouse lines causing RP [23]. Moreover, the results suggest that photoreceptor cell death does not occur cell-autonomously but proceeds through cell‒cell interactions.

However, there are still unanswered questions for retinal degeneration. For example, it appears that there are other unidentified signals involved in the progression of the disease besides those induced by endothelin. Furthermore, the specifics of cell-to-cell communication during the onset of retinal degeneration have yet to be clarified. Additionally, while the expression analysis revealed changes in multiple genes, identifying the specific cells in the retina where the alterations occur is difficult due to its cellular diversity. Therefore, precise determination of altered gene expression within the retina requires analysing individual cells rather than the whole tissue.

We now took advantage of single-cell expression profiling to examine the genetic changes in two *Prom1*-KO retinas—one subject to light exposure and the other unexposed. Gene expression analysis was conducted at the single-cell level, revealing the downregulation of IGF signalling in photoreceptor cells and astrocytes under light-stimulated conditions. The combination of Endothelin inhibitor treatment and compensation of IGF1 signalling substantially restored the retinal phenotypes. We will further demonstrate that mTOR, which mediates IGF signalling, is crucial for the survival of photoreceptors and the maintenance of retinal tissue integrity.

## Results

### The retina at the age of 11 days expresses genes specific to the retina, and susceptibility to light stimulation is observed in the ***Prom1*** mutant

We first investigated expression in 11-day-old *Prom1*+/−  retinas to determine whether functional cells exist in the retina early after birth. At this age, mice have not opened their eyes, and the retina has never been exposed to any stimulation from the periphery, including light exposure; therefore, the phenotypes can be significantly dependent on their genotypes.

**Fig. 1:**
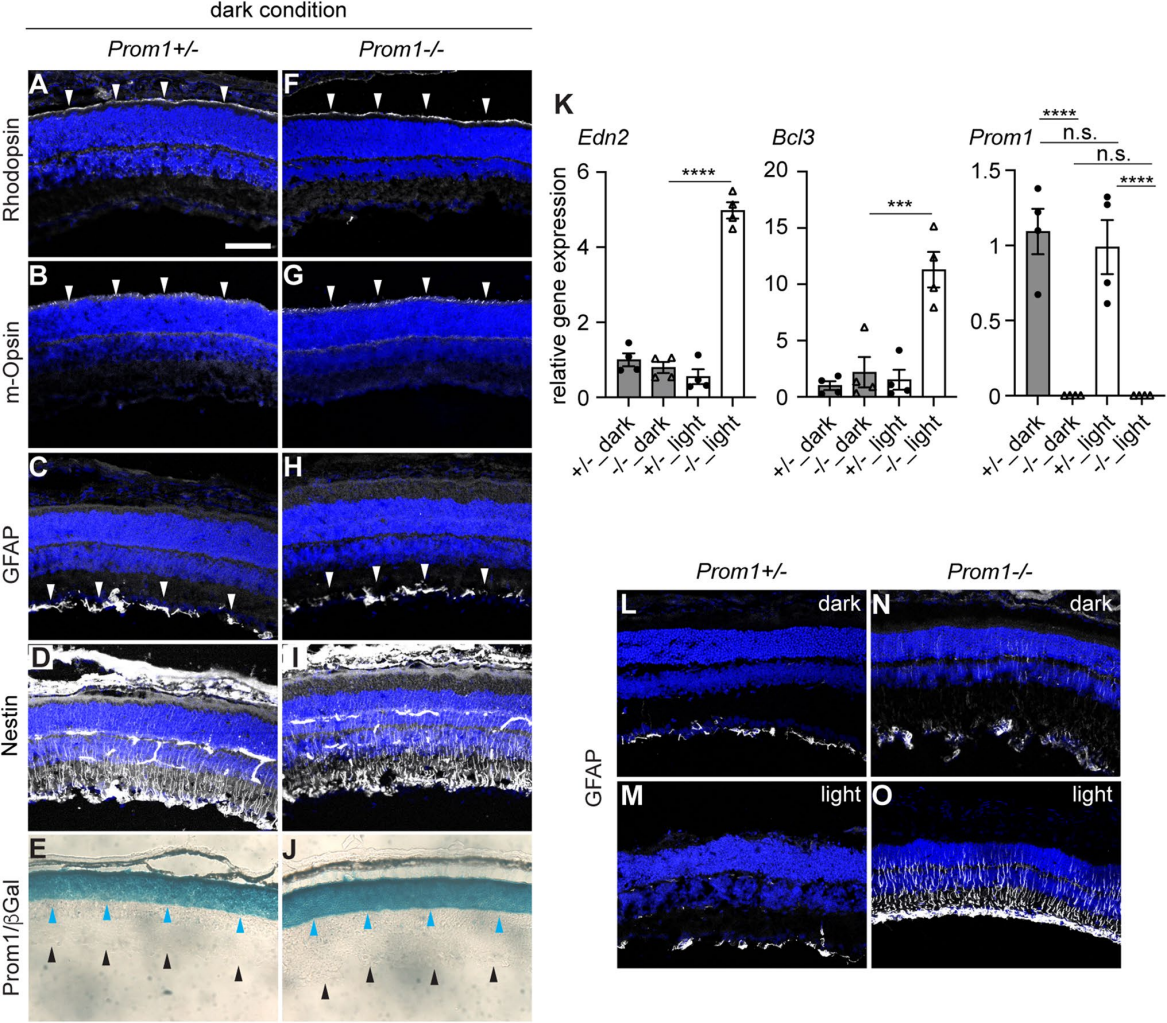
Retinal gene expression is intact in the *Prom1−/−* retina but is vulnerable to light stimulation. Immunofluorescence with RHO (**A**, **F**), m-Opsin (**B**, **G**), GFAP (**C**, **H**), and Nestin (**D**, **I**) and β-galactosidase staining (β-Gal; **E**, **J**; blue arrowheads) in 11-day *Prom1*+/− (**A**–**E**) (**A**–**D**) and *Prom1−/−* (**F**–**J**) retinas. Expression is indicated by white arrowheads, and DAPI is represented by blue signals (**A**–**D**, **F**–**I**). The inner borders of ONL and the retina are indicated by blue and black arrowheads, respectively (**E**, **J**). **K** RT‒qPCR analysis of *Edn2*, *Bcl3* and *Prom1* in the retinas of *Prom1*+/− and *Prom1−/−* mice reared in the dark or with light stimulation (*n* = 4 for each condition). GFAP distribution in the 3-weels-old *Prom1*+/− (**L**, **M**) and *Prom1−/−* (**N**, **O**) retinas that did not (**L**, **N**) or did (**M**, **O**) undergo light stimulation at 11 days of age was analysed by immunofluorescence. Scale bar in (**A**) for (**A**–**J**, **L**–**O**) = 100 μm

Immunostaining revealed the presence of outer segments of rod and cone photoreceptor cells by the rhodopsin (RHO) (Fig. 1A) and m-Opsin (Fig. 1B) signals, respectively, located outside the ONL. Detection of the glial markers GFAP (Fig. 1C) and Nestin (Fig. 1D) was additionally observed. Prom1 expression was further confirmed through β-galactosidase staining (Fig. 1E). Therefore, functional cells have developed before the eyes open.

We next examined whether these cells also exist in the homozygotic *Prom1* mutants. The retina was overall indistinguishable from the heterozygotic mutant (Fig. 1A–E), and the distribution of the proteins above was unchanged (Fig. 1F–J), suggesting that retinal development was unaffected by the loss of Prom1 function.

In our previous study [6], we demonstrated that retinas devoid of the *Prom1* gene are highly susceptible to light stimuli, leading to aberrant activation of glial cells called gliosis. To determine whether early postnatal retinal cells respond to light exposure as in the juvenile and adult stages, we forcibly opened the eyes of the *Prom1* heterozygotic and homozygotic mice, exposed them to 15,000 lx light stimulation for 3 h, and returned them to the dark environment overnight to ensure the accumulation of mRNAs. The whole retina was then extracted, and the gene expression was compared with those reared in the continuous dark environment by reverse-transcription and quantitative polymerase chain reaction (RT‒qPCR).

As a result, we found upregulated gene expression of *endothelin-2* (*Edn2*), encoding endothelin peptide (ET-2), which negatively affects retinal homeostasis [6, 18], and *B-cell lymphoma 3* (*Bcl3*), which is involved in cell survival [24], under the light condition in the *Prom1−/−* retina but not in the *Prom1*+/−  retina under either light or dark conditions (Fig. 1K). Thus, light stimulation did not affect the *Prom1* expression level (Fig. 1K).

In addition, while the *Prom1*+/−  retina did not exhibit any aberrantly activated Müller glial cells upon temporal light stimulation (Fig. 1L, M), a number of GFAP signals extending into glial cells were found in the light-stimulated *Prom1−/−* retina, with only a slight increase in the dark-reared *Prom1−/−* mice (Fig. 1N, O). Thus, glial cells are also reactive to the signals induced by light exposure in the *Prom1*-deficient mice.

Together, the results suggest that retinal tissue robustness against light-induced stress depends on the existence of Prom1, and that the *Prom1−/−* retina is primed to light stimulation as early as postnatal day 11.

### Single-cell expression profiling identified the cells and genes whose expression was altered by light exposure

We next sought to identify the genes and cells directly affected by light stimulation. For this purpose, we conducted single-cell gene expression profiling on retinas that were exposed to light stimulation. To minimise possible individual differences caused by the rearing environment, we employed two *Prom1*-KO mice that were born from the same mother and processed them in the same way as in the previous RT‒qPCR analysis (Fig. 1K). The retinal cells were then dissociated, and ten thousand cells were subjected to single-cell RNA sequencing (scRNA-seq) to compare the gene expression with another *Prom1−/−* mouse reared in persistent dark conditions (see Material and Methods for details).

**Fig. 2:**
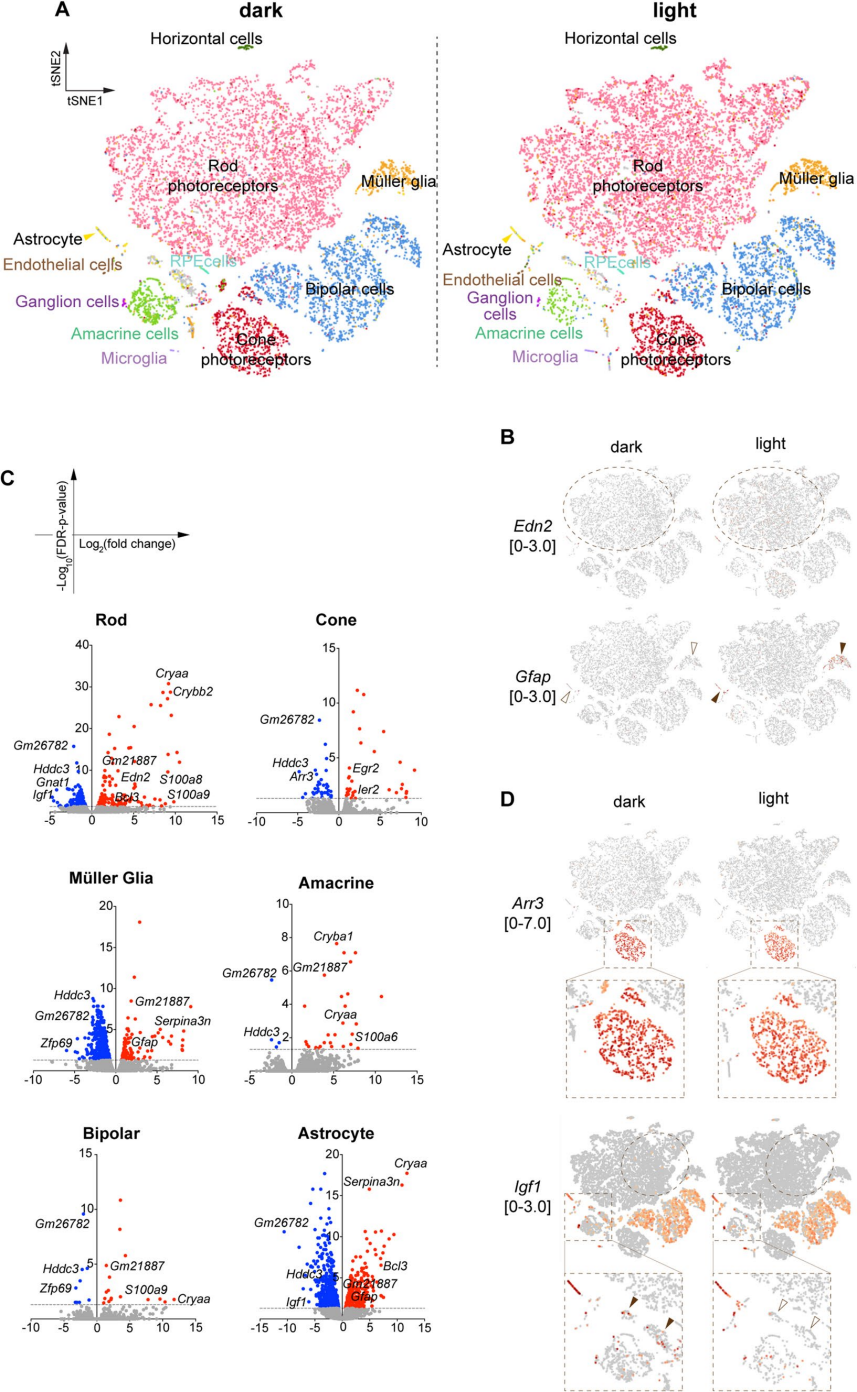
Gene expression altered by light stimulation in the *Prom1−/−* retina. **A** A t-SNE representation of scRNA-seq from retinas reared in the dark environment and exposed to light stimulation as categorised into 11 types of cells by the genes representatively expressed in specific clusters. **B** Heatmap representation of *Edn2* and *Gfap* expression. The areas surrounded by dotted ovals are where gene expression is changed. **C** Volcano plots showing the fold changes (horizontal axis) and *p* values (vertical axis) of the genes in each category. **D** Heatmap representations of *Arr3* and *Igf1.* In **B** and **D**, filled and open arrowheads indicate the presence and absence of expression, respectively. The group of cells expressing *Igf1* in the dark are indicated by dotted circles. The cells for *Igf1* expression are shown in large plots for visibility

From two sets of transcriptome data produced from both dark and light conditions, the whole tendency of gene expression was visualised by t-distributed stochastic neighbour embedding (t-SNE) expansion (Fig. 2A, Supplementary Fig. S1A). Overlaying spots did not exhibit apparent changes in the distributions of the cells, suggesting that the two samples were comparable in developmental and maturing conditions (Supplementary Fig. S1A).

Next, the cell types were categorised into 11 groups by the genes specifically expressed in each cell type (Table 1, Supplementary Fig. S1B). For instance, rod cells were sorted by the unique expression of *RHO*, *Sag*, *Pdc*, *Gngt1*, and *Rp1* (55.3% in the dark and 59.8% in the light), and cone cells were sorted by *Opn1sw* (8.6% in the dark and 7.9% in the light). The other types of retinal cells were likewise identified by the genes expressed uniquely in the indicated cells (Supplementary Fig. S1B). The ranking of the population in the retina was unchanged overall between the two conditions (Table 1), suggesting that cell death or selective exclusion of specific cell types had not yet started.

**Table 1:** The categorisation of the cells and changes in gene expression

Cell type	Marker genes	Number of cells		% of cells		Number of upregulated genes	Number of downregulated genes
		Dark	Light	Dark	Light		
Rod photoreceptor	*Sag, RHO, Pdc, Gngt1, Rp1*	5376	6812	55.3	59.8	145	126
Bipolar cell	*Nrxn3, Vsx2*	1733	1780	17.8	15.6	17	9
Cone photoreceptor	*Opn1sw*	835	894	8.6	7.9	31	37
Müller cell	*Crym*	478	757	4.9	6.6	106	433
Amacrine cell	*Gad1*	444	193	4.6	1.7	28	4
Astrocyte	*Igfbp5*	118	102	1.2	0.9	308	672
Horizontal Cell	*Lhx1, Prox1*	52	48	0.5	0.4	0	0
Endothelial Cell	*Cldn5*	28	29	0.3	0.3	2	1
Microglia	*C1qa*	20	36	0.2	0.3	0	0
RPE cell	*RPE65*	19	24	0.2	0.2	0	0
Ganglion cell	*Sncg, Pou4f1*	19	12	0.2	0.1	0	0
Others	–	593	700	6.1	6.1	–	–
Total	–	9715	11387	100.0	100.0	–	–

The genes expressed in each cell type and the number of categorised cells

In rod photoreceptor cells, 271 genes (145 upregulated genes and 126 downregulated genes) were changed in expression, with p values lower than 0.05 (Table 1). This gene list included *Edn2* as an upregulated gene, confirming that the endothelin peptide ET-2 is produced and emanates from rod photoreceptor cells [6, 18] (Fig. 2B, C). Moreover, the *Gfap* and *Serpina3n* genes, whose expression is responsive to ET-2 [25], were enriched in Müller glia and astrocytes by light stimulation (Fig. 2B, C), suggesting that the glial reaction had already started [26]. In contrast, no genes were altered in RPE cells (Table 1). This observation suggests that photoreceptor cells, rather than RPE cells, were the earliest cell type affected by light stimulation at the onset of RP.

We further found that bipolar and rod photoreceptor cells exhibited the upregulation of *S100a8* and *S100a9* (Fig. 2C), encoding calcium- and zinc-binding proteins activated upon neuroinflammation [27]. We also found that a number of crystallin genes (*Cry*) were upregulated, including *α-crystallin (Crya),* whose expression has been shown to be upregulated by stress [28], under light conditions in most retinal cell types (Fig. 2C). Thus, the *Prom1−/−* retinal cells exhibit the stress response to light stimulation by inducing different sets of genes, and the response occurs not only in the photoreceptor cells where Prom1 is mainly expressed (Fig. 1E) but also in the surrounding cells.

We were also aware that some of the genes essential for visual functions were downregulated by light stimulation in the *Prom1−/−* retina. In photoreceptor cells, *Arrestin3* (*Arr3*) and *G Protein Subunit Alpha Transducin 1* (*Gnat1*), both of which play critical roles in phototransduction, decreased (Fig. 2C, D), suggesting that *Prom1* forms a gene regulatory network with the other genes for retinal functions and is an upstream gene.

Overall, single-cell expression profiling successfully identified the cells and genes that respond to light stimulation at the earliest stage of retinal degeneration.

### *Igf1* expression in rod photoreceptors and astrocytes is downregulated in light-stimulated *Prom1* mutants and affects ribosomal protein S6 phosphorylation

Among the genes altered by light stimulation, we sought to focus on extracellular molecules, because changes in their expression levels can impact surrounding cells and regulate the condition of the entire retina. Along this line, we particularly became interested in *Igf1*, as *Igf1* was found to be downregulated upon light stimulation mainly in rod photoreceptors and astrocytes (Fig. 2C, D, Supplementary Fig. S2A, B), while the expression did not significantly change in other cell types (Supplementary Fig. S2A, B). Some cells, which were not classified into any categories but were assumed to have similar characteristics to astrocytes, also exhibited downregulation of *Igf1* expression (Fig. 2D; filled and open arrowheads).

IGF1, encoded by *Igf1*, has been shown to have a neurotrophic effect on neurons, including photoreceptor cells, by activating prosurvival and antiapoptotic signal pathways [29, 30]. Consistently, a lack of IGF signalling by knocking out the IGF1 receptor gene leads to photoreceptor degeneration [31]. Therefore, we speculated that the decrease in IGF1 is one of the triggers for photoreceptor degeneration.

**Fig. 3:**
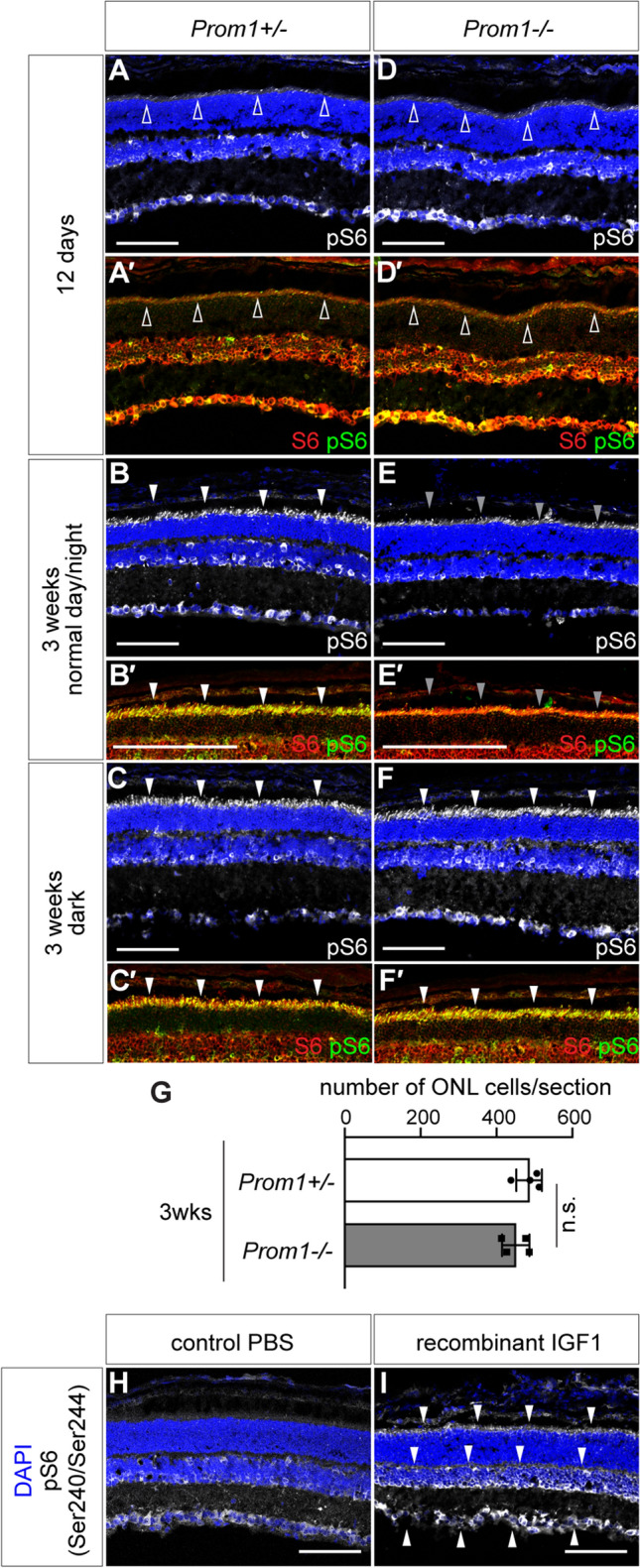
Decrease in the phosphorylated S6 protein accounts for the loss of *Igf1* expression. **A**–**F**′ The presence of pS6 (Ser240/Ser244) at the inner segment of the photoreceptor layer is dependent on light stimulation under *Prom1* mutation. Immunofluorescence with pS6 (Ser240/Ser244) in 12-day-old (white in (**A**, **D**); green in (**A**′, **D**′)), 3-week-old under normal day/night (white in (**B**, **E**); green in (**B**′, **E'**)), and 3-week-old under dark condition (white in (**C**, **F**); green in (**C**′, **F**′)) of *Prom1*+/−  (A-C') and *Prom1−/−* (**D**–**F**′) retinas. Staining with total S6 is indicated by red signals (**A**′, **B**′, **C**′, **D**′, **E**′, **F**′). DAPI staining is shown in blue, and the presence, absence and decreased expression of pS6 is indicated by white filled, open and grey filled arrowheads, respectively. **G** The number of DAPI-positive cells in the ONL in *Prom1*+/− and *Prom1−/−* mice at 3 weeks (3 weeks) of age (*n* = 4 each). **H**, **I** IGF1 induces the phosphorylation of the S6 protein. Three-hour retinas injected with PBS (**H**) or recombinant IGF1 (**I**) were stained with the pS6 antibody. DAPI staining is shown in blue, and ectopic expression is indicated by arrowheads. Scale bars = 100 μm

Given that the IGF signal can be mediated by mTOR activation [32], we first sought to address the activation of the downstream protein. For this purpose, we asked whether the ribosomal protein S6 is phosphorylated at Ser240 and Ser244 (hereafter denoted as pS6), because these residues are directly phosphorylated by the S6 kinase p70S6K via the activation of the IGF/mTOR signal [33]. In the *Prom1* heterozygotic littermates at 12 days old, pS6 was detected in the ganglion cell layer (GCL) and at border areas of the inner nuclear layer (INL) (Fig. 3A), but only a little at the photoreceptor layer, while the total S6 protein was detectable (Fig. 3A'; open arrowheads). On the other hand, at 3 weeks, the pS6 signals immediately outside of the ONL were found, in addition to the same cells at 12 days (arrowheads in Fig. 3B, B′). Moreover, the signals were localised to the inner segment of the photoreceptor cells, as the signal was complementary to that of RHO and s-Opsin, which are localised to the outer segment (Supplementary Fig. S2C, D). The pS6 signal at the inner segment of the photoreceptor cells was found even in the retina where the mice were reared in the continuous dark environment at 3 weeks (Fig. 3C, C′), suggesting that phosphorylation occurs in an age-dependent manner but is independently regulated from the dark/light environment where the mice are reared.

In the *Prom1−/−* retinas, pS6 was localised in areas similar to those of the *Prom1*+/−  retinas at 12 days of age (Fig. 3D, D′); phosphorylation was found in the GCL and at the border of INL, but not in the photoreceptor cells. In contrast, at 3 weeks old, while the localisation in the GCL and INL was detectable as that in the *Prom1*+/−  mice, significantly less pS6 was detected outside the ONL (Fig. 3E, E′; grey arrowheads). On the other hand, the *Prom1−/−* retinas reared in the dark exhibited an abundance of pS6 similar to that found in *Prom1*+/−  retinas (Fig. 3F, F′). In these retinas, the number of ONL cells was comparable in both genotypes (Fig. 3G), suggesting that the decrease in pS6 was unlikely due to the secondary effect caused by the alteration in the number of photoreceptor cells.

These findings suggest that phosphorylation of S6 in photoreceptor cells normally increases as growth progresses; however, light stimulation on the *Prom1−/−* retina caused a decrease in pS6 positivity of the photoreceptor cells. Considering that IGF/mTOR signalling resides as an upstream regulatory system for S6 phosphorylation, this observation is consistent with a light-dependent suppression of *Igf1* expression levels.

To verify that S6 phosphorylation can be induced by IGF, we injected recombinant IGF1 protein into the vitreous of 12-day-old wild-type mice and examined the localisation of pS6 in the retina three hours after injection. IGF1 injection, but not control PBS, induced ectopic S6 phosphorylation, particularly at the inner segment of photoreceptors and at the border area of INL (Fig. 3H, I; white arrowheads). Thus, the cells are competent to respond to the IGF signal and its downstream S6 protein can be phosphorylated.

Together, the data suggest that *Igf1* is one of the downregulated genes upon light stimulation in the retina and is concomitant with the phosphorylation of the S6 protein.

### Persistent IGF1 expression improves photoreceptor cell survival

We next asked whether the compensation of the IGF signal in the *Prom1−/−* retina recovers retinal survival and functions. To ensure sustained IGF expression, we conducted adeno-associated virus (AAV) infection into the retina using intravitreal injection and investigated the ameliorating effects of the infection.

**Fig. 4:**
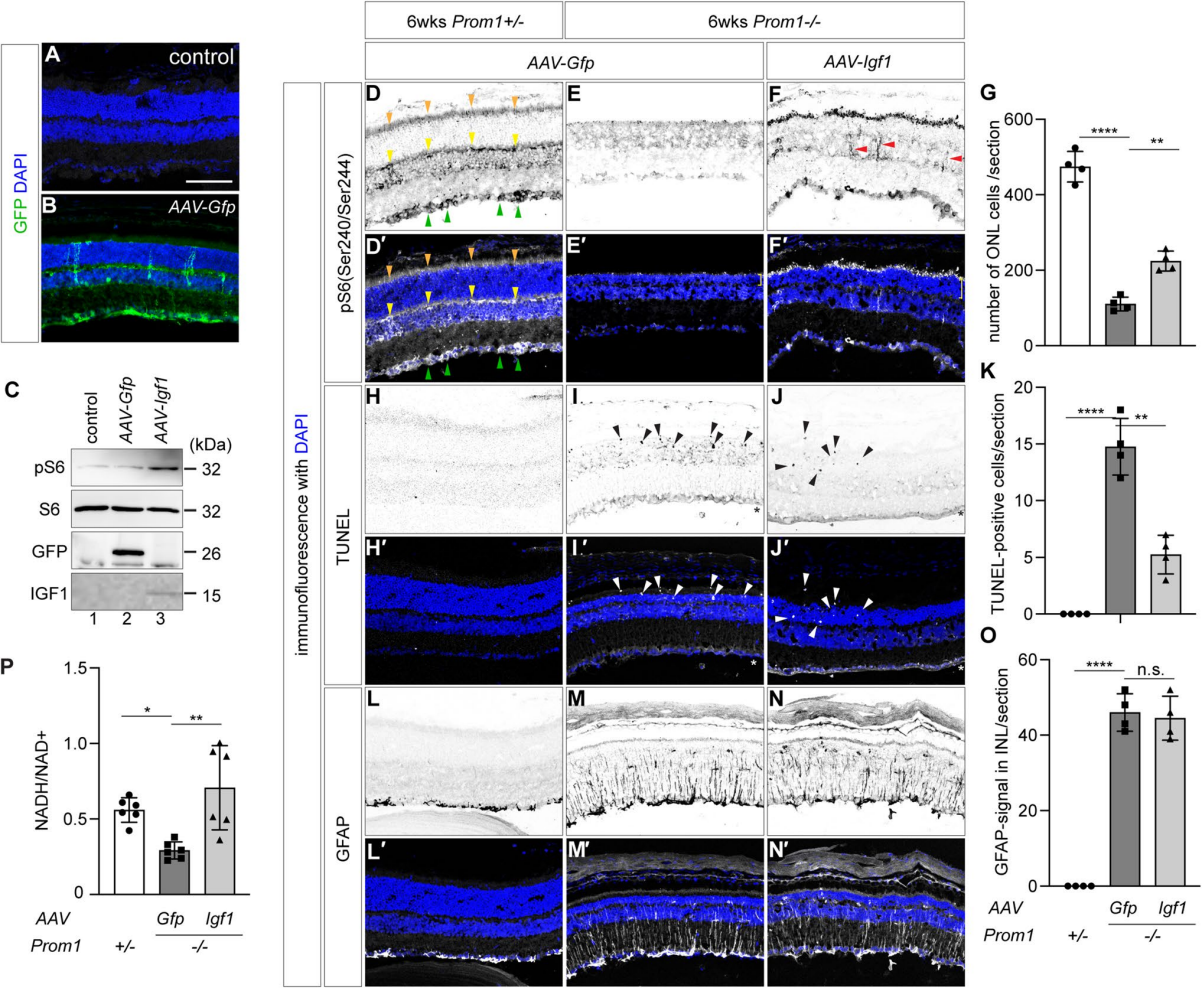
Overexpression of IGF by AAV promotes cell survival in the *Prom1−/−* retina. The control retinal section (**A**) or the retinal section infected with *AAV-Gfp* at 2 weeks of age (**B**), extracted at 6 weeks of age. GFP (green in **B**) was detected by immunofluorescence with DAPI staining (blue signals in (**A**, **B**). **C** Western blot analysis of pS6, S6, GFP and IGF1 was performed on the control retinal cell extract (lane 1) or those infected with *AAV-Gfp* (lane 2) or *AAV-Igf1* (lane 3) to verify the infection. Note that the endogenous IGF1 was not detectable. **D**–**O** Effects of single AAV infection in 2-week-old conveying *GFP* (**D**, **D′**, **E**, **E′**, **H**, **H′**, **I**, **I′**, **L**, **L′**) or *Igf1* (**F**, **F′**, **J**, **J′**, **N**, **N′**) genes in the *Prom1*+/−  **(D**, **D′**, **H**, **H′**, **L**, **L′**) and *Prom1−/−* (**E**, **E′**, **F**, **F′**, **I**, **I′**, **J**, **J′**, **M**, **M′**, **K**, **K′**, **N**, **N′**) mice extracted at 6 weeks of age. pS6 (Ser240/Ser244) (D-F′), TUNEL (**H**–**J′**) and GFAP (**L**–**N′**) expression was analysed by immunofluorescence (**D**–**F**′, **L**–**N′**) or TUNEL analyses (**H**–**J′**). In (**D**, **D′**, **E**, **E′**), the signals of pS6 at the inner segment of photoreceptors (orange arrowheads), INL (yellow arrowheads) and GCL (green arrowheads) are indicated. Red arrowheads in (**F**) indicate ectopic pS6 signals. Representative TUNEL signals are indicated by black (**I**, **J**) and white (**I′**, **J′**) arrowheads, whereas asterisks at GCL indicate non-specific staining. Quantification of the signals. The numbers of DAPI in ONL (**G**) and TUNEL-positive cells (**K**) and GFAP signals crossing the INL (**O**) are indicated. For (**G**, **K**, **O**), four samples were analysed for each genotype and treatment. **P** Infection with *AAV-Igf1* improves glucose metabolism. The NADH/NAD^+^ ratios of the cell extracts from *Prom1*+/−  and *Prom1−/−* with *AAV-GFP* (control) or with *AAV-Igf1* were measured (*n* = 6 for each specimen)

To validate AAV infection, we prepared 6-week *Prom1*+/−  retinas that were either uninfected (Fig. 4A) or infected with *AAV-Gfp* at 2 weeks of age (Fig. 4B). As a result, we found GFP signals broadly in the retina, with strong signals at GCL and Müller glial cells caused by the infection (Fig. 4B).

To verify the infection of *AAV-Igf1*, we performed a western blot analysis on the *Prom1*+/−  retina infected with AAVs conveying *Gfp* (control) or *Igf1*. We found that pS6 was upregulated by *AAV-Igf1*, but not by *AAV-Gfp*, at 2 weeks post infection, confirming that *AAV-Igf1* properly activated IGF signalling (Fig. 4C).

Based on these validations, we infected *AAV-Gfp* and *AAV-Igf1* into the *Prom1*+/−  or *Prom1−/−* retinas and investigated the phenotypes caused. In 6-week-old retinas (4 weeks postinfection), pS6 was localised at the GCL (Fig. 4D, D′; green arrowheads), INL (Fig. 4D, D′; yellow arrowheads), and photoreceptor layer (Fig. 4D, D′; orange arrowheads). While the signals were almost undetectable in the *Prom1−/−* retina upon infection with *AAV-Gfp* (Fig. 4E, E′), infection with *AAV-Igf1* showed partially rescued signals of pS6 (Fig. 4F, F′), with some ectopic signals (Fig. 4F; red arrowheads) detectable. Notably, a higher number of cells were found at the ONL in the *Prom1−/−* retina upon infection with *AAV-Igf1* (Fig. 4E′, F′, G), suggesting that IGF signalling has a protective effect against retinal degeneration caused by a lack of Prom1 function.

We reasoned that the increased number of ONL cells was caused by reduced programmed cell death and conducted a terminal deoxynucleotidyl transferase dUTP nick end labelling (TUNEL) assay. We found several apoptotic cells in the *Prom1−/−* retina upon infection with control *AAV-Gfp* (Fig. 4I, I′, K), which was never detected in the *Prom1*+/−  retina (Fig. 4H, H′, K) under the same experimental conditions; however, infection with *AAV-Igf1* significantly decreased the number of TUNEL-positive cells (Fig. 4J, J′, K). Therefore, programmed cell death was perturbed by the IGF signal.

We further investigated the effect of IGF1 on glial activation. A larger number of GFAP-positive glial cells reaching the INL were detected in the *Prom1−/−* retinas than in the *Prom1*+/−  retinas (Fig. 4L–M′,O). However, this number did not decrease upon infection with *AAV-Igf1* (Fig. 4N, O), suggesting that IGF signalling does not have an ameliorating effect on gliosis and further suggesting that IGF acts independently of neuroinflammation signals.

IGF/mTOR signalling has been shown to activate aerobic glycolysis, a metabolic process in which glucose is converted to pyruvate. Through several transfer reactions, the oxidised form of nicotinamide adenine dinucleotide (NAD^+^) is reduced to NADH, and adenosine triphosphate (ATP) is produced [34]. Based on this knowledge, we asked whether aerobic glycolysis was activated upon infection with *AAV-Igf1* and measured the NADH/NAD^+^ ratio in 6-week-old retinas.

We found that the retinal cell extracts from *Prom1−/−* mice showed a lower ratio of NADH/NAD^+^ than those from *Prom1*+/− mice (Fig. 4P). In contrast, the *AAV-Igf1-*infected *Prom1−/−* retinas exhibited a restoration of NADH/NAD^+^ levels, suggesting that the reduction reaction of NAD^+^ to NADH was activated by the overexpression of IGF1 (Fig. 4P).

Together, IGF1 has blocking activity against programmed cell death and thereby protects the retina, including mainly photoreceptor cells, without attenuating GFAP signalling.

### The combination of IGF1 overexpression and blockade of endothelin signalling improves retinal function in *Prom1−/−* mice

Previous studies have shown that administering endothelin receptor antagonists ameliorates gliosis and suppresses photoreceptor cell death [6, 18]. Therefore, we next attempted to understand the functional relevance of endothelin receptor antagonists and IGF on gliosis or retinal functions.

**Fig. 5:**
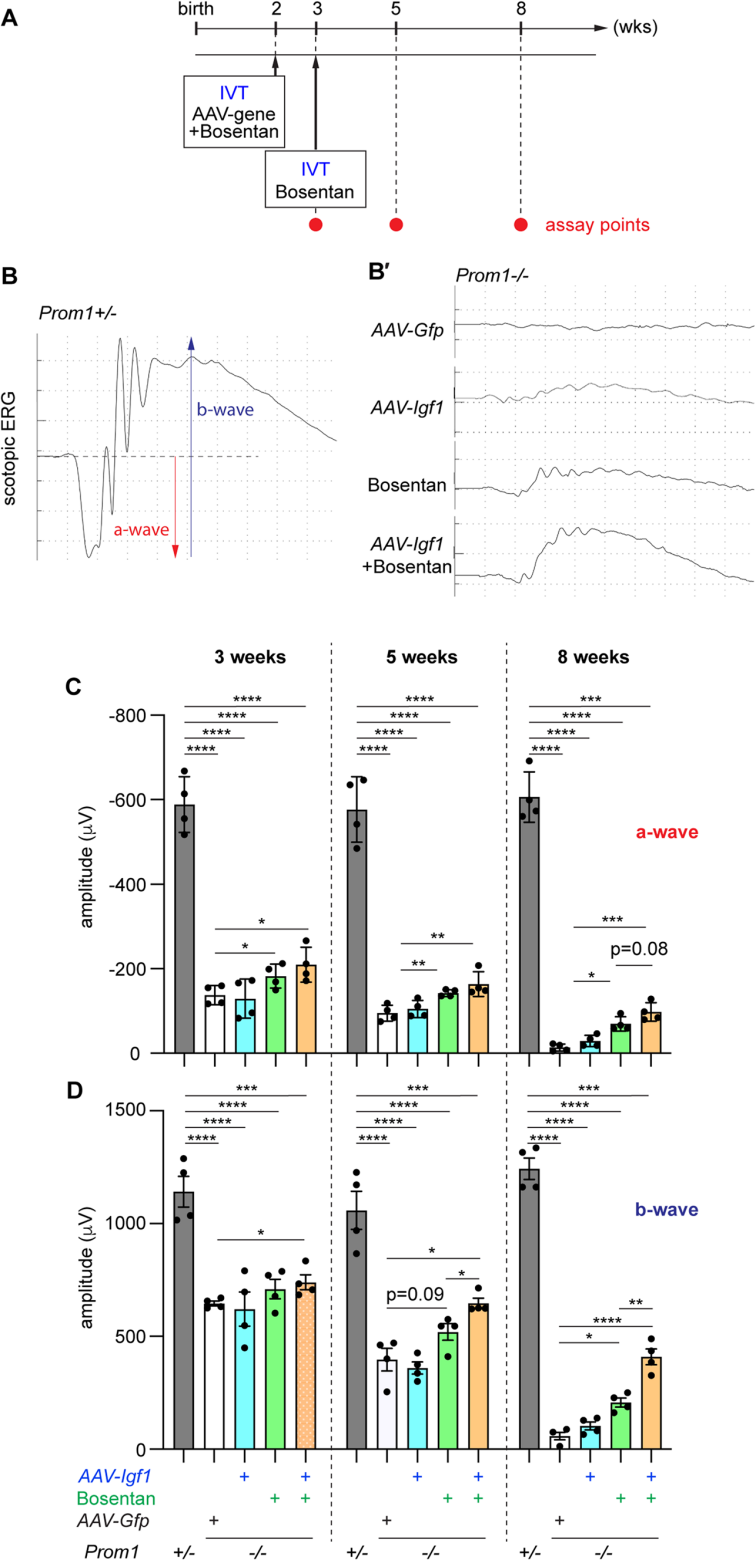
Introduction of IGF1 in addition to treatment with bosentan improves retinal function in *Prom1−/−* mice. **A** Experimental schedule for AAV infection, drug administration, and ERG measurement. **B**, **B**′ Representative peak images of each treatment obtained at 8 weeks of age. The a-wave (red) and b-wave (dark blue) are indicated. Quantification of the amplitudes of a- (**C**) and b-waves (**D**) upon each treatment. 3-, 5- and 8-week-old mice either from *Prom1*+/− (grey bars) or *Prom1−/−* mice were treated with *AAV-Gfp* (control; white bars), *AAV-Igf1* (blue bars), bosentan (green bars) or bosentan + *AAV-Igf1* (orange bars). 4 eyes were measured from each condition. Note that the scores for a-waves (**C**) are negative values

Herein, we used bosentan as the endothelin antagonist, which targets two endothelin receptors, EdnrA and EdnrB, with a similar binding affinity (35–38) and is clinically used to treat pulmonary arterial hypertension [39]. In this study, injections were performed intravitreally to ensure a local effect (Fig. 5A).

To validate the effect of bosentan, we first administered vehicle (control; dimethyl sulfoxide (DMSO)) or bosentan to 2-week-old *Prom1*+/−  or *Prom1−/−* retinas. Vehicle or bosentan was additionally administered 1 week after the first injection (Fig. 5A for the injection schedule). The bosentan-administered *Prom1−/−* retinas showed significantly fewer GFAP-positive (Supplementary Fig. S3A–C′) and TUNEL-positive cells (Supplementary Fig. S3D–F′) than the control retinas treated with control DMSO. Therefore, bosentan is effective in blocking the excess activation of Müller glial cells and programmed cell death.

We next sought to address the correlation between the suppression of cell death and functional improvement and carried out electroretinography (ERG). In this physiological assay, two peaks of the initial negative (a-wave: reflecting the function of the photoreceptor cells) and the following positive peak (b-wave: mainly detecting the function of horizontal and Müller glial cells) evoked by a light pulse can be evaluated (Fig. 5B). *Prom1*+/−  retinas showed comparable a- and b-wave amplitudes at 3, 5, and 8 weeks of age, suggesting that the 3-week-old retinas are already functionally similar to those of mature individuals (Fig. 5C, D; dark grey bars). On the other hand, in the *Prom1−/−* retina, both a- and b-waves were significantly lower than those in the *Prom1*+/−  retina at 3 weeks of age (Fig. 5C, D; light grey bars), confirming that retinal function had started to malfunction before this stage, although the number of retinal cells was still comparable to that in the heterozygotic littermates. Therefore, even though the cells have not evidently degenerated at 3 weeks of age, the characteristics have changed, and cells are not as functional as in the control retinal cells. The ERG response decreased over time in the *Prom1−/−* retinas, and the waveforms were almost undetectable at 8 weeks of age, suggesting that the *Prom1−/−* mice were completely blind at 8 weeks of age (Fig. 5B′–D).

Next, we asked whether the IGF signal exhibits an improving effect on this function and infected *AAV-Igf1*. However, significant improving effects were not found at any age tested, at least in our experimental regime (Fig. 5C, D; blue bars). We next tried bosentan to examine the effect of the endothelin blockade. Compared with control *AAV-Gfp*-treated *Prom1−/−* retinas, bosentan-administered mutants exhibited significantly improved a-wave amplitudes at 3 weeks of age. This trend persisted at 5 and 8 weeks of age (Fig. 5C, D; green bars), and the b-wave amplitude was also found to be improved at 8 weeks of age, confirming that bosentan improves retinal function. We further examined the combination of bosentan and *AAV-Igf1*, and noticeably, we found that all amplitudes of the a- and b-waves were significantly greater than those of the control *Prom1*-homozygotic mutants (Fig. 5C, D; orange bars).

Therefore, while the single treatment with the endothelin signalling blocker on the degenerating retina exhibits improvements in retinal functions, the simultaneous manipulation of two signals, endothelin and IGF, exhibits superior protective effects.

### mTOR-mediated signalling is needed for photoreceptor survival

Among several intracellular downstream pathways induced by IGF [30, 40], we asked whether the mTOR-mediated branch is involved in retinal homeostasis and tissue integrity. As the null mutation of the mTOR gene causes early embryonic lethality [41], we generated the drug-inducible and neural and glial- and temporal-specific knockout line (*Nestin-CreERT2; mTOR f/f*), where the mTOR gene was conditionally removed.

We crossed homozygotic floxed *mTOR* mice [42] with mice expressing Cre recombinase fused with the oestrogen receptor driven by the *Nestin* promoter [43]. This line enabled the nuclear translocation of Cre recombinase upon tamoxifen administration.

**Fig. 6:**
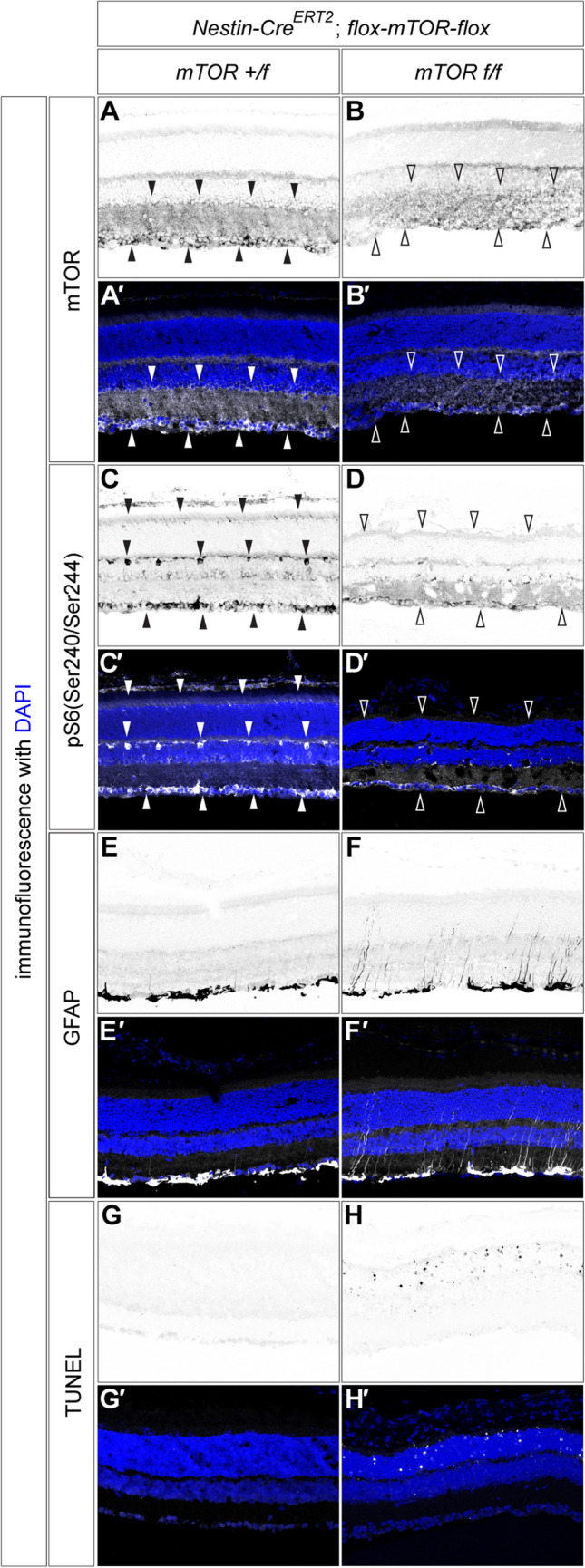
mTOR signalling is essential for retinal tissue integrity. The mice with *mTOR* + */f* (**A**, **A′**, **C**, **C′**, **E**, **E′**, **G**, **G′**) or *mTOR*  *f/f* (**B**, **B′**, **D**, **D′**, **F**, **F′**, **H**, **H′**) harbouring *Nestin-Cre*^*ERT2*^*;mTOR* + */f* were injected with Tamoxyfen at 2 weeks old and analysed when they reached 6 weeks of age by immunofluorescence with mTOR (**A**–**B′**), pS6 (**C**–**D′**), GFAP (**E**–**F′**) and TUNEL (**G**–**H′**). Filled and open arrowheads indicate present and lost signals, respectively

We intraperitoneally administered tamoxifen to *Nestin-CreERT2; mTOR f/*+ and *Nestin-CreERT2; mTOR f/f* mice to induce recombination at 2 weeks of age, and the retina was subjected to analysis at 4 weeks post-administration when the mice reached 6 weeks of age. The partial attenuation of the mTOR gene was validated by immunofluorescence (Fig. 6A–B′; filled and open arrowheads for presence and absence, respectively), and accordingly, the activation of the target substrate pS6 was lost as well (Fig. 6C–D′). Moreover, evident upregulation of GFAP expression was found in mTOR-conditional knockout mice (Fig. 6E–F′), suggesting that the loss of mTOR induces neuroinflammation. An increased number of apoptotic cells was found in *Nestin-CreERT2; mTORf/f* mice (Fig. 6G–H′), further demonstrating that the signals mediated by mTOR are necessary for retinal cell survival.

Together, the data suggest that mTOR signalling is needed for the maintenance of retinal functions and are consistent with the notion that IGF signalling plays an important role in cell survival.

## Discussion

### Retinal degeneration progresses through intercellular communication

In this study, we conducted single-cell gene expression profiling in the *Prom1−/−* retina exposed to short light stimulation and identified the earliest responsive cells and genes. We identified *Igf1* as one of the key factors whose expression was altered by light stimulation. Combined treatment with an endothelin receptor blocker and sustained IGF1 expression improves retinal survival and function during the progression of RP.

RP is a type of retinal dystrophy that results in the degeneration of photoreceptor cells and/or RPE. The causative RP genes are, in most cases, expressed in either or both types of cells [44]. Accordingly, as the photoreceptor and RPE cells collaboratively regulate the redox state of the retinoid [2], complications with either the photoreceptors or the RPE cells eventually result in similar phenotypes of visual dysfunctions. As Prom1 is mainly expressed in photoreceptor cells (Fig. 1E), it is reasonable to conclude that rod and cone photoreceptor cells are the cell types most severely affected by light stimulation, with changes in the expression of several genes (Table 1). However, in addition to photoreceptor cells, our scRNA-seq analysis identified astrocytes and Müller glial cells as the cell types where a number of genes were altered (Table 1). These cells were presumably affected secondarily by the complications occurring in the photoreceptor cells, considering that Prom1 is expressed in photoreceptor cells (Fig. 1E). The present study successfully identified the genes and cells affected at the onset of retinal degeneration. Importantly, we demonstrate that that photoreceptor degeneration occurs via cell‒cell communication with the surrounding cells, rather than cell-autonomously.

Such intercellular communication and the activation of glial cells have been observed in other RP mutants [18, 45], and one of the strong candidates involved in this interaction is ET-2, produced by the *Edn2* gene. *Edn2*/ET-2 is a common inflammatory factor that activates glial cells in the central nervous system [46], and light stimulation triggers *Edn2* expression in rod photoreceptor cells (Fig. 2B, C) [6, 18]. In glial cells, the responsive genes *Gfap*, *Serpina3n*, and *S100a8/a9*, which are inflammation-related and/or stress-responsive, are induced [25, 26, 47] (Fig. 2B, C). These reactive glial cells, in turn, release neurotrophic factors to promote the survival of surrounding cells [48] and phagocytose dead photoreceptors [49], thereby playing a positive role in retinal homeostasis.

However, one serious paradox of excessive activation of glial cells is that they induce the glial scar, which is a cell aggregate that causes a spatial obstruction to the retina, and retinal function is rather perturbed [50]. Moreover, the activation of ET-2 exerts profound effects on the development and homeostasis of the retinal vasculature, perturbing vascular development [23], inducing the constriction of retinal venules [6, 22], and injuring the blood‒retinal barrier [51]. Targeting gliosis is, therefore, a potential clinical strategy to delay disease progression and ameliorate associated symptoms [52, 53], and ET-2 is a strong target for this purpose.

Upregulation of the endothelin pathway has been found in a number of other mutant retinas causing RP, including those of rd10 mice, where the *phosphodiesterase 6b* (*Pde6b*) is mutated [18], endothelin signal antagonists are adequate to ameliorate the degenerating phenotype [18, 54], and diabetic model db/db mice carrying a mutation in the leptin receptor gene [55]. Therefore, it is highly assumable that similar mechanisms are common during RP, and endothelin receptor antagonists are effective in delaying the symptoms. The blockade of endothelin signalling is, therefore, a common strategy to ameliorate the retinal phenotypes of retinitis pigmentosa.

It is evident that the blockade of the endothelin signal at the earliest step of phenotypic emergence is critical and effective for reducing gliosis, as a reduction can be found even after a long period of antagonist injection (Fig. 5; Supplementary Fig. S3). This is probably because the mutant retina is more sensitive to light stimulation at its young stages, and induces a higher level of *Edn2* when the photoreceptor cells start to deteriorate. However, retinal function does not completely correspond to the reduction in gliosis, as retinal function cannot be sufficiently restored (Fig. 5). Thus, more strategies in addition to blocking gliosis are necessary to improve retinal function.

This study suggests that the IGF signal induced by the *Igf1* gene is a possible signal that compensates for retinal function (as discussed in detail in the next section). Additionally, several genes were found to be altered by light stimulation. For instance, *Hddc3* and *Zfp69*, whose expression is downregulated under light conditions, are involved in ferroptosis [56] and insulin sensitivity during diabetes [57, 58], respectively (Fig. 2C). Notably, the downregulation of *Zfp69* is also found in db/db mice [59], which suggests that metabolic disorder occurs at the onset of retinopathy in the *Prom1−/−* retina. In addition, *Gm26782* and *Gm21887*, encoding long noncoding RNAs (lncRNAs), were changed in some of the retinal subtypes (Fig. 2C). The functions of these genes could be explored during photoreceptor degeneration.

However, unlike *Edn2*, *Hddc3*, *Zfp69* and the lncRNA genes were not commonly found in scRNA-seq analyses with other gene mutants [60–62]. These differences may reflect the diversity in the severity of the phenotypes in each gene mutation.

### Light stimulation downregulates IGF1 signalling in the *Prom1−/−* retina

IGF1 is a versatile signalling molecule involved in several biological events, including differentiation [63, 64], proliferation [65], cell motility [66], metabolism [67] and survival [30]. In particular, IGF, ciliary neurotrophic factor (CNTF), brain-derived neurotrophic factor (BDNF), and fibroblast growth factors (FGFs) act as neurotrophic factors in the central nervous system [68, 69], including the injured retina [30, 69], and exhibit a rescue effect on retinal cells. For instance, IGF1/IGF1R signalling plays a role in retinal vascularisation and growth [70, 71]. Conversely, attenuation of the *Igf1* gene [29, 30, 72] leads to blockade of autophagy and causes neuroinflammation [29]. Moreover, the knockout of the *Igf1r* gene [31] causes vision impairment by altering the metabolic processes of retina, fatty acid, and phosphoinositide [31].

By means of single-cell RNA sequencing, we found that *Igf1* gene expression was downregulated in rod photoreceptor cells and astrocytes by light stimulation (Fig. 2C, D). At the stage of the sequencing analysis, there was no expression of pS6 expression found in either the *Prom1*+/− or *Prom1−/−* photoreceptor cells, presumably because *Igf1* expression was too low to be detected and differences in expression could not be assessed either by Igf1 or pS6 expression. In the 3-week-old retina, the localisation of pS6 in photoreceptor cells became evident in the *Prom1*+/− retina (Fig. 3B, B′). However, in the *Prom1−/−* retina, light stimulation inhibited the localisation of pS6 in photoreceptor cells (Fig. 3E, E′).

Furthermore, the decrease in pS6 in photoreceptor cells was found to be specific to light-stimulated conditions (Fig. 3F, F′). We referred to our previous transcriptome analysis of the whole retina [6] and found that *Igf1* and *Pkm*, encoding enzymes involved in glycolysis [34], were downregulated in the *Prom1−/−* retina. This observation is consistent with the decrease in the *Igf1* gene found in the present study. One possible mechanism for this phenotype is that the properties of the cells producing IGF1, either rod photoreceptors or astrocytes, were altered by the inflammatory responses, making IGF1 production difficult. The administration of *Igf1* in the retina by AAV (Fig. 4F) successfully restored retinal survival (Fig. 4J, K).

While pS6 expression strongly suggests the downregulation of Igf signal in the *Prom1−/−* retina, we have not succeeded in experimentally and directly detected the Igf1 gene/protein expression in either wild-type and *Prom1−/−* mice. Consequently, it is not possible to quantitatively evaluate the range of influence of IGF1 signals in the retina. Establishing a transgenic or knock-in line with a tagged version of IGF1, such as IGF1-GFP, would facilitate the visualisation of the IGF1 protein and overcome that limitation.

### IGF1 signal complements the endothelin blockade in maintaining retinal function

Photoreceptor cells are where aerobic glycolysis is active [34], and IGF signalling has been shown to play an essential role in this metabolic process [31]. Therefore, it is reasonable to assume that the IGF signal was partially inactivated in degenerating photoreceptor cells, and the compensation of IGF1 reactivates glycolysis and affects the NADH/NAD^+^ ratio (Fig. 4P).

IGF evokes several intracellular downstream signals, including those mediated by PI3K/AKT, mTOR, and ERK [73]. Our analysis revealed that mTOR-mediated signalling is, at least in part, essential for retinal survival (Fig. 6). mTOR signalling has been shown to generally play essential roles in cell survival [32], and our findings are in good agreement with this notion (Fig. 4J, K). Moreover, mTOR has been shown to be essential for autophagy [74]. Therefore, the involvement of mTOR in IGF-induced cell survival provides a reasonable explanation for retinal survival.

However, one caveat for IGF activity is that there are also reports demonstrating the negative effects of IGF1 on the retina, where sustained IGF1 expression by a transgene induces retinal cell death and gliosis [75]. In addition, local upregulation of IGF1 has been shown to trigger blood‒retinal barrier breakdown, and accordingly, humans with retinopathy with marked gliosis exhibited upregulation of IGF1R expression [76]. However, in these studies, the analysed stages were elderly, more than 3 months old in mice, and older than 80 years old in humans. Therefore, one possible interpretation for these seemingly contradictory phenotypes is that while cells require IGF activity, the excess level of IGF1 expression causes intense glycolysis, leading to earlier exhaustion. Therefore, to ensure the positive aspect of IGF signalling for retinal survival, it is necessary to control the IGF intensity quantitatively, and the establishment of its administration method is awaited.

Our functional analysis suggested that the sustained expression of IGF1 by AAV combined with treatment with endothelin receptor blockers tends to improve retinal function (Fig. 5). Considering that IGF activates aerobic glucose metabolism to maintain photoreceptor survival in a cell-autonomous manner, whereas endothelin receptor antagonists block neuroinflammation and glial hyperactivation, these two signals function in a complementary, but not redundant, manner to ameliorate the degenerative phenotype of the retina.

In exploring therapeutic methods for RP, one of the prominent methods currently established is the transplantation of retinal pigmented epithelium cells differentiated from stem cells into the degenerated area [77]. Gene transfer into patients, so-called gene therapy [78–80], is also in the clinical stage. While regenerative techniques and gene compensation are expected to be fundamental therapeutic methods, there is still a need for more generally applicable treatments that target the causative gene. The method we propose in this study meets these requirements, and targeting molecules acting in the extracellular space is particularly useful to ensure drug accessibility.

## Materials and methods

### Animals and treatments

Generation of the *Prom1*-mutant mice, where the locus has been replaced with the *LacZ* gene, was described elsewhere [81], and the stock can be found at the following site (https://large.riken.jp/distribution/mutant-list.html). The conditional knockout line of *mTOR* (*Mtor* < *tm1.2Koz* > */J*) was purchased from Jackson Laboratory. Cre-ERT2 driven by the *Nestin* promoter (*Tg(Nes-cre/ERT2)5.1 Imayo*) [43] was distributed by the Riken BioResource Center (RBRC05999; https://mus.brc.riken.jp/ja/order) with the permission of Itaru Imayoshi.

Mice were usually reared at 20 °C with a 12 h-dark/light cycle with ad libitum access to food and water. To examine the effect of light stimulation, the pups were reared in persistent dark condition until the experiment, the eyelids of young mice whose eyes were still closed were incised with a scalpel, and the eyes were forcibly opened. Pups were dilated with topical application of 0.5% tropicamide and 0.5% phenylephrine (Santen; Mydrin-P^®^) to efficiently introduce light stimulation into the retina. The mice were reared under LED light at 15,000 lx for 3 h.

### Single-cell RNA sequencing analysis

For scRNA sequencing analysis, two male pups with the homozygous *Prom1* mutation from the same dam were obtained. One of them was exposed to light stimulation for 3 h at 11 days of age as described in the previous section and immediately returned to the cage where the siblings were reared to recover overnight. The mice were sacrificed the next day and the extracted retinas were dissociated using the Papain Dissociation System (Worthington Biochemical Corporation; #PDS). We successfully isolated approximately 2 × 10^5^ cells from one retina with a viability of 80%.

The Chromium Next GEM Single Cell 5′ Kit v2 (10 × Genomics) was used for scRNA-seq library construction according to the manufacturer's instructions. Libraries were sequenced on an Illumina NovaSeq 6000 at a read length of 28 × 90 to obtain a minimum of 20,000 reads per cell for 10,000 cells. The resulting raw data were processed using Cell Ranger 6.0.0 (10 × Genomics).

Gene reads were obtained from 10,311 and 12,091 cells under dark and light conditions, respectively. Of these, 596 (5.8%; dark) and 704 (5.8%; light) cells were excluded from the analysis objects, because these cells had 1000 or fewer unique molecular identifiers (UMIs), 256 or fewer genes detected, or more than 10% of the genes were of mitochondrial origin. Next, each cell was categorised based on the representative genes specifically expressed in each cell type [82, 83], with the order being RPE cells, endothelial cells, microglia, horizontal cells, ganglion cells, Müller glia, cone photoreceptors, bipolar cells, astrocytes, amacrine cells and rod photoreceptor cells. The gene list with the fold changes and *p* values of each gene can be found in Supplementary Table S1.

### RNA, protein extraction and expression analysis

For RT‒qPCR, RNA and cDNA were prepared by using NucleoSpin^®^ RNA (MACHEREY‑NAGEL; 740955) and PrimeScript RT Master Mix (Takara, RR036), respectively. Quantitative PCR was performed on a CFX qPCR machine (Bio-Rad), and the primer sequences are listed in Supplementary Table S2. Amplification data were analysed using the comparative Ct method, and the expression of each gene was normalised to that of *RhoA*.

For western blotting, retinas infected with *AAV-Gfp* and *AAV-Igf1* were harvested and snap frozen with liquid nitrogen. They were dissociated with extraction buffer containing 50 mM Tris–Cl (pH 7.8), 0.1% NP-40, 1 mM EDTA and 150 mM NaCl and sonicated three times for 10 s each. 20 μg of the extracts were resolved by sodium dodecyl sulfate–polyacrylamide gel electrophoresis (SDS-PAGE) on 12.5% (for pS6, S6 and GFP) and 15% (for IGF1) polyacrylamide and transferred to a Polyvinylidene fluoride or polyvinylidene difluoride (PVDF) membrane (Millipore; iseq00010). The membranes were primary and secondary antibodies. Signals were developed using LAS4000 (Fujifilm).

### Tissue analysis

For immunofluorescence, the eyeball was extracted at the indicated stage and fixed with paraformaldehyde. In 30 min, the lens was enucleated to encourage the penetration of the fixative and incubated for 30 more minutes. The tissues were incubated with 15% sucrose overnight and embedded in OCT compound (Sakura). The sectional samples were prepared in a Tissue Polar cryostat (Sakura fintek, Japan) at a thickness of 10–12 µm. The samples were incubated with primary antibodies overnight at 4 °C and subsequently with secondary antibodies for 2 h at room temperature. Nuclei were visualised by 4′,6-diamidino-2-phenylindole (DAPI). The antibodies used in this study are listed in Supplementary Tab. S2.

β-galactosidase staining was performed with staining solution containing 1 mM MgCl_2_, 5 mM K_4_[Fe^II^(CN)_6_], and 5 mM K_3_[Fe^III^(CN)_6_] in PBS supplemented with 1 mg/ml 5-bromo-4-chloro-3-indolyl-β-d-galactopyranoside (X-gal).

For TUNEL staining, sectioned specimens were incubated for 2 min with 0.1 M citric acid (pH 6.0) and were incubated in the reaction buffer containing in (Merck; S7105) 1xTdT reaction buffer, 1 mM CoCl_2_, Terminal Transferase recombinant (Roche; 03333566001), and the signals were detected with anti-Digoxin antibody (Supplementary Table S2). Cells positive for TUNEL, found mainly in ONL and its surrounding areas, were manually counted on one picture, and more than three images were taken from each experimental condition. A typical image is shown as a figure.

The NADH/NAD^+^ ratio was determined using a CycLex NAD^+^/NADH Colorimetric Assay Kit (MBL; CY-1253V2) according to the manufacturer's instructions, and the absorbance at 450 nm was measured using an iMark microplate reader (Bio-Rad).

### Preparation and injection of AAV

To generate AAVs carrying *Gfp* (control) or *Igf1*, we prepared *pAAV-EGFP* (for control; distributed by Addgene (#32395)) [84] and *pAAV-IGF1*, which was generated by replacing *Gfp* with the *Igf1* gene. These AAVs produce the AAV2 serotype and the genes were driven by the cytomegalovirus (CMV) promoter. The AAVs were generated by transfection of one of the *pAAV* vectors with *pHelper* and *pRC2-mi342* packaging vectors (TaKaRa #632608) into HEK293 cells, and the AAVs were harvested 4 days post-transfection using the AAVpro^®^ Purification Kit Midi (TaKaRa #6675). The titres of the AAV were determined by PCR and were stocked as 5 × 10^9^/μl in PBS.

### Intravitreal injections into mice

Mice were anaesthetised with three types of mixed anaesthetic agents by intraperitoneal injection. A mixed anaesthetic was prepared with 0.75 mg/kg medetomidine, 4.0 mg/kg midazolam, and 5.0 mg/kg butorphanol. In addition, the cornea was anaesthetised with 0.4% oxybuprocaine hydrochloride ophthalmic solution (Santen; Benoxil). 10 μg of bosentan monohydrate (Tokyo Chemical Industry; B5118; in DMSO), 1 μg of human IGF1 (PeproTech; AF-100-11; in PBS), or 10^9^ genome copies of AAV were prepared alone or in mixtures and were introduced as 1 μl into the vitreous body of both eyes of each animal with a 35 G needle (Saito Medical Instruments). After AAV treatment, 0.5% levofloxacin ophthalmic solution (Santen; Cravit) was applied to the ocular surface to prevent infection, and 0.75 mg/kg atipamezole was intraperitoneally injected to reverse anaesthesia.

### Functional analysis of the retina by ERG

ERG was performed by using the Neuropack S3 (Nihon Kohden, Co. Ltd.), along with the LED Visual Stimulator LS-100 (Mayo Corporation) and the Hemisphere Stimulator (Mayo Corporation). All manipulations were performed under dim red light. Mice were adapted to the dark environment for at least 1 h and were anaesthetised as described above. Tested animals were kept on a heating mat throughout the procedure to maintain body temperature. Pups were dilated with a topical application of 0.5% tropicamide and 0.5% phenylephrine. Gold ring electrodes were placed on the corneal surfaces, and needle electrodes were inserted as ground and reference electrodes on the nose and tail, respectively. Responses to a 3162 cd/m^2^ white light flash (1 ms) were amplified, filtered, and recorded [31, 85]. The a-wave amplitudes were measured from the baseline to the trough of the a-wave, and the b-wave was measured from the trough of the a-wave to the peak of the b-wave.

### Conditional knockout of the *mTOR* gene

Mice carrying the *flox-mTOR* gene [42] were purchased from Jackson Laboratory (IMSR_JAX:011009), and either heterozygotic or homozygotic mutants were intraperitoneally administered 1 mg of (Z)-4-hydroxytamoxifen (Abcam ab141943) at 2 weeks of age and were harvested at 6 weeks of age by perfusion fixation [86].

### Image processing and statistics

Images were captured using LSM980 confocal microscopes (Zeiss) and processed using Photoshop software (Adobe). Figures were prepared using Illustrator (Adobe) and Prism ver.10 (GraphPad). Numerical information in each graph is provided in Supplementary Table S3. Differences were evaluated using either a two-tailed Student’s *t* test (for comparisons between two groups) or one-way analysis of variance (ANOVA; for comparisons between more than two groups). Statistical comparisons with *p* < 0.05 were considered significant. *p* values (*p < 0.05; **p < 0.01; ***p < 0.001; ****p < 0.0001) are indicated in each graph.

### Supplementary Information

Below is the link to the electronic supplementary material.

Supplementary file1 (PDF 3731 KB)

Supplementary file2 (XLSX 7705 KB)

Supplementary file3 (XLSX 10 KB)

Supplementary file4 (XLSX 14 KB)

## Data Availability

The raw data for the single-cell RNA sequencing have been deposited at the DNA Data Bank of Japan (DDBJ) with the deposition number DRA016296.
