# Supplementary material for: Combination of blockade of endothelin signalling and compensation of IGF1 expression protects the retina from degeneration
Source: Cell Mol Life Sci. 2024 Jan 22;81(1):51. doi: 10.1007/s00018-023-05087-x (PMC10803390; doi:10.1007/s00018-023-05087-x)
Supplement: Supplementary file 1 — Supplementary file1 (PDF 3731 KB) [file 18_2023_5087_MOESM1_ESM.pdf]

Supplementary Materials

Combination of blockade of endothelin signalling and compensation of IGF1 expression protects the retina from degeneration

Naoya Shigesada[1], Naoya Shikada[1], Manabu Shirai[2], Michinori Toriyama[3], Fumiaki Higashijima[4], Kazuhiro Kimura[4], Toru Kondo[5], Yasumasa Bessho[1], Takuma Shinozuka[1] and Noriaki Sasai[1,*]

1. Division of Biological Science, Nara Institute of Science and Technology, Ikoma, 630-0192, Japan
2. Omics Research Center (ORC), National Cerebral and Cardiovascular Center, Suita, Osaka 564-8565, Japan
3. Department of Biomedical Chemistry, School of Science and Technology, Kwansei Gakuin University, Sanda 669-1337, Japan
4. Department of Ophthalmology, Graduate School of Medicine, Yamaguchi University, Ube 755-0046, Japan
5. Division of Stem Cell Biology, Institute for Genetic Medicine, Hokkaido University, Sapporo 060-0815, Japan

Corresponding author
* Noriaki Sasai (noriakisasai@bs.naist.jp)

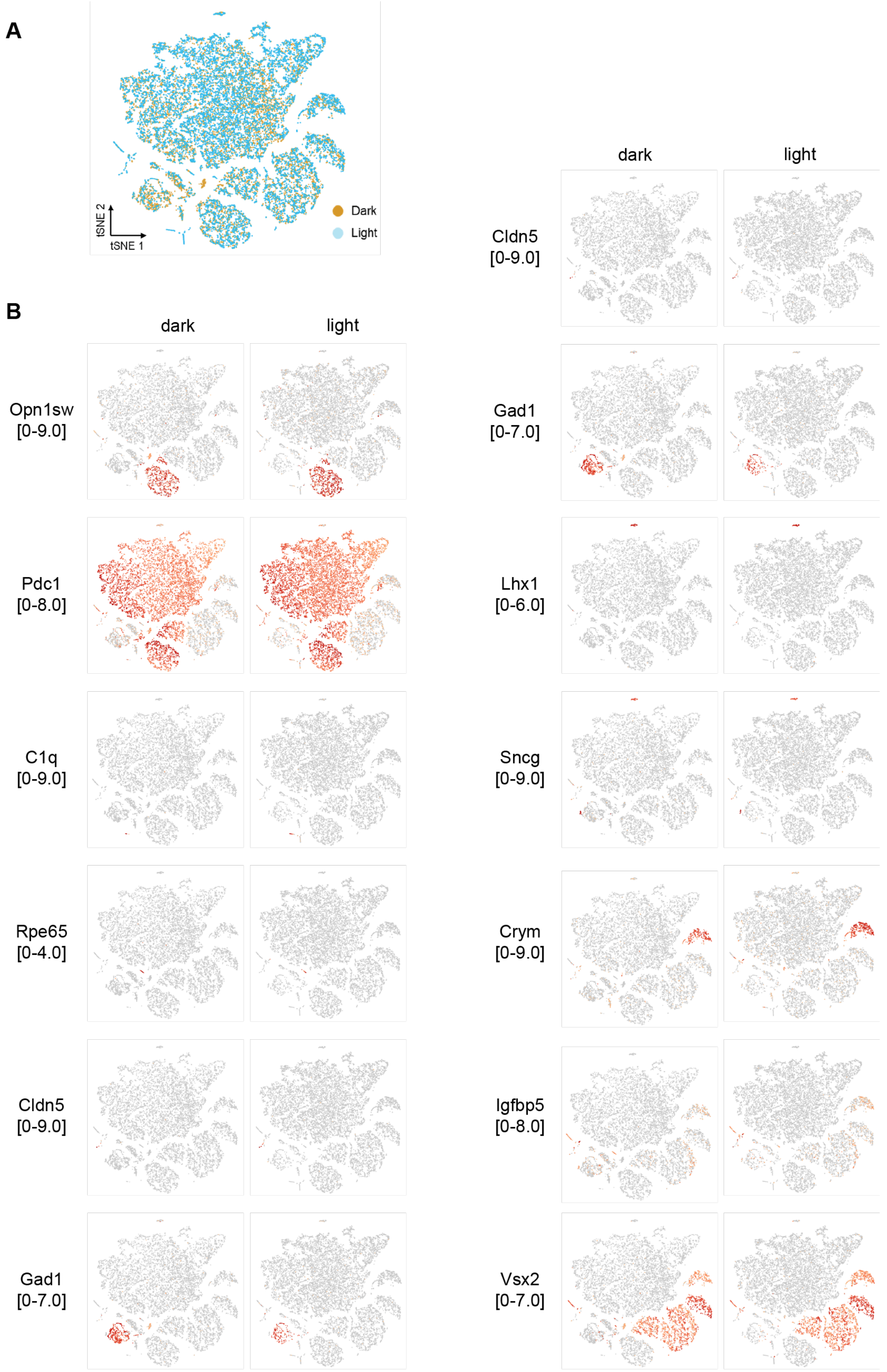

Supplementary Fig. S1 Single-cell transcriptome data produced from the dark and light conditions, in relation to Fig. 2

(A) Overlayed t-SNE representation of the dark and light conditions. (B) Heatmap of the gene expression, characterising the specific cell types, in relation to Fig. 2 and Tab. 1.

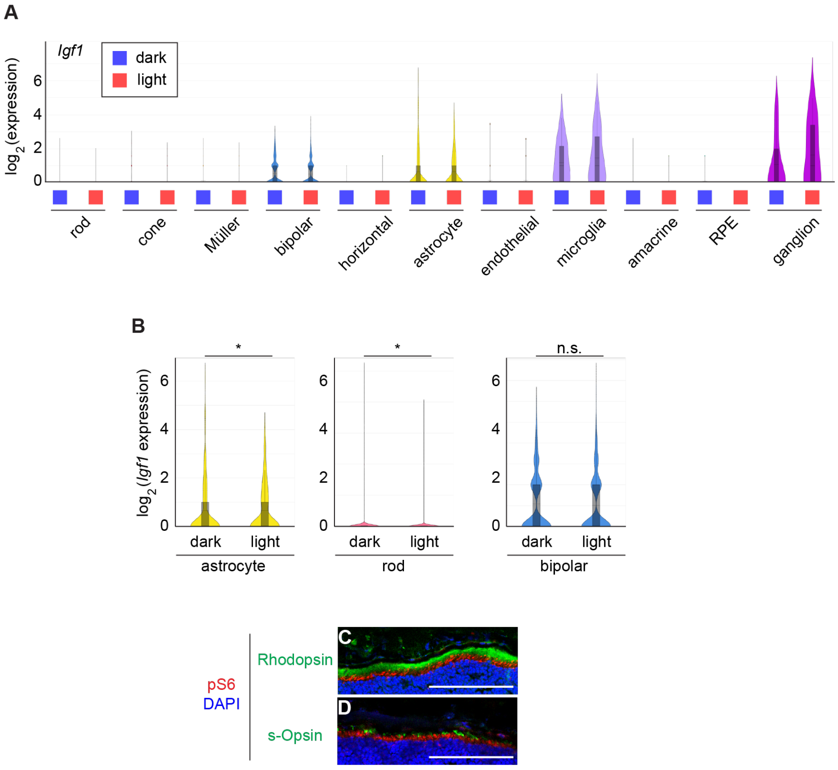

Supplementary Fig. S2 *Igf1* expression by cell type and localisation of pS6 in the photoreceptor cells. (A,B) Violin plots of *Igf1* expression in all eleven cell types (A), and those focused on astrocytes, rods and bipolar cells, as extracted from the scRNA-seq analysis. (C,D) The localisation of pS6 is complementary to that of rhodopsin and s-Opsin. Co-immunofluorescence of Rhodopsin and pS6 (C) or s-opsin and pS6 (D). Scale bars = 100 µm.

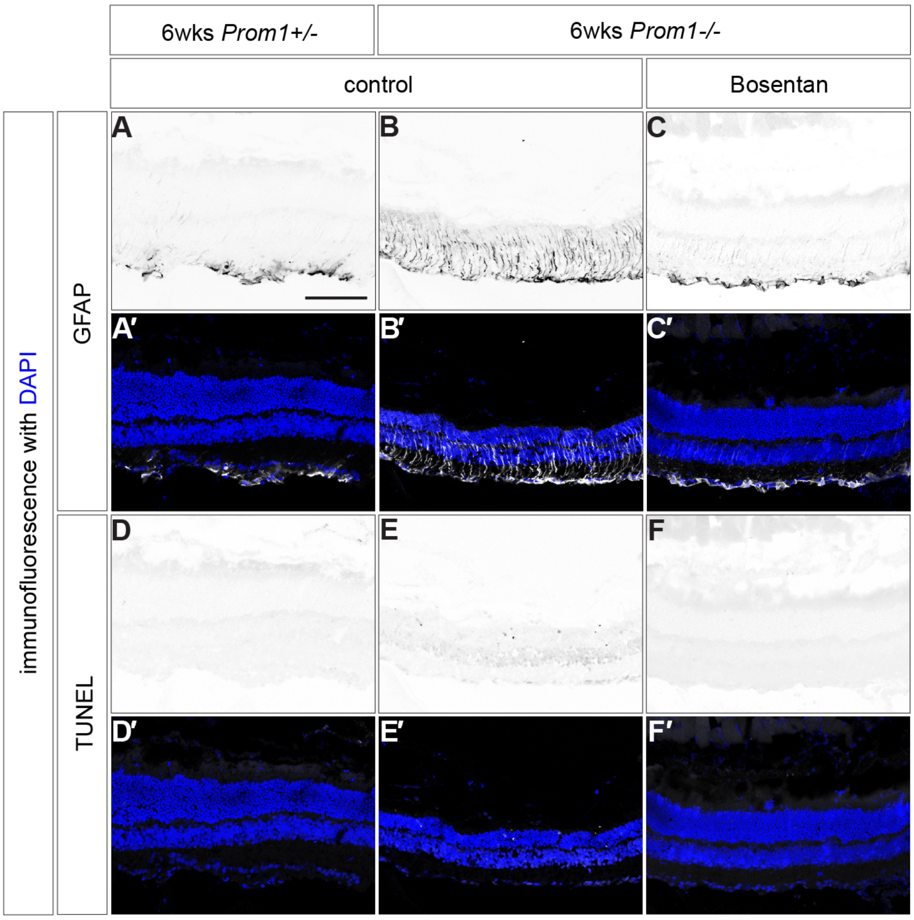

Supplementary Fig. S3 Bosentan decreases GFAP-positive glial cells and supresses programme cell death. Intravitreal injections of control vehicle (A,A',B,B',D,D',E,E') or bosentan (C,C',F,F') were given on *Prom1+/-* (A,A',D,D') or *Prom1-/-* (B,B',C,C',E,E',F,F') retinas twice at 2 and 3-week-old ages. Treated retinas were harvested at 6-week-old. GFAP (black in (A,B,C) and white in (A',B',C')) and TUNEL (black in (D,E,F) and white in (D',E',F')) signals were detected by immunofluorescence with DAPI staining (blue signals in (A',B',C',D',E',F')). Scale bar in (A) = 100 µm for all panels.

Supplementary Tab. S1 Comparison of the gene expression in each cell type, in relation to Fig. 2C.

Supplementary Tab. S2 List of the antibodies and primer sequences used in this study.

Supplementary Tab. S3 Numerical information on the bar graphs.
